# Distinguishing pain profiles among individuals with long COVID

**DOI:** 10.3389/fresc.2024.1448816

**Published:** 2024-10-18

**Authors:** Laura Tabacof, Maanas Chiplunkar, Alexandra Canori, Rebecca Howard, Jamie Wood, Amy Proal, David Putrino

**Affiliations:** ^1^ Cohen Center for Recovery from Complex Chronic Illness, Department of Rehabilitation and Human Performance, Icahn School of Medicine at Mount Sinai, New York, NY, United States; ^2^ Rusk Rehabilitation, Department of Physical Medicine and Rehabilitation, New York University Grossman School of Medicine, New York, NY, United States; ^3^ Polybio Research Foundation, Boston, MA, United States

**Keywords:** long COVID, pain, neuropathic, cognition, cognitive function, rehabilitation, quality of life

## Abstract

**Background:**

For many people with long COVID (LC), new-onset pain is a debilitating consequence. This study examined the nature of new-onset pain and concomitant symptoms in patients with LC to infer mechanisms of pain from the relationships between pain and health-related factors.

**Methods:**

Pain and other symptoms were evaluated in 153 individuals with LC using the Self-Administered Leeds Assessment of Neuropathic Symptoms and Signs, EuroQoL Visual Analog Scale, and Quality of Life in Neurological Disorders. The relationships between pain and patient factors were analyzed using Chi Square and independent *t*-tests.

**Results:**

20.3% of individuals who reported new-onset pain had neuropathic pain, which was associated with lower quality of life and higher rates of cognitive dysfunction compared to those with non-neuropathic pain. Other symptoms were similar between groups, however heart-related symptoms were more prevalent in individuals with neuropathic pain and mood swings were more prevalent for individuals with non-neuropathic pain.

**Conclusions:**

Characterizing the relationships between NP and quality of life in individuals with LC can aid in developing better clinical management strategies. Understanding the associations between NP and cognitive dysfunction provides the imperative foundation for future studies further examining the pathophysiological mechanisms underlying pain development in LC.

## 1 Introduction

Long COVID (LC) is a post-acute infection syndrome that is estimated to affect over 65 million people worldwide (1). People with LC experience a wide array of symptoms that can persist for months to years after their acute SARS-CoV-2 infection, with over 200 distinct LC symptoms identified (2). The majority of individuals with LC experience between five and eight symptoms that detrimentally influence overall quality of life (3), making LC a complex condition to manage. New-onset musculoskeletal pain is frequently reported in people with LC, but the nature of this pain has not been well-characterized in previous work, with few mechanistic explanations for why people with LC may be experiencing debilitating pain (4).

Pain is a complex and challenging research topic. When exploring new-onset chronic pain associated with LC, an important starting point would be to first differentiate between pain that may be neuropathic in origin compared to that which is non-neuropathic. Similar mechanisms to nociplastic pain may contribute to the development of pain in LC (5), therefore the initial distinction is crucial as potential interventions may differ widely. A recent case control study suggested that L-acetyl-carnitine in combination with physical exercise reduces musculoskeletal pain and improves quality of life in patients with LC (6). However, neuropathic pain (NP) in LC has been hypothesized to be caused by increased proliferation of cytokines mediated by angiotensin-converting enzyme 2; this subsequently allows the entrance of the virus into the peripheral nerve cell itself, triggering a release of tumor necrosis factor alpha2. While both small and large fiber nerves are impacted, recent findings indicate that small-caliber, lightly myelinated, or unmyelinated nerves are particularly prone to harm and the subsequent development of NP pain (7). However, there is still very little work that aims to disambiguate new-onset pain subtypes that present in LC.

In this brief report, we examined the nature of new-onset pain reported by a convenience sample of 153 people with LC and explored potential associations between LC symptoms and pain. Uncovering symptoms that frequently occur alongside new-onset pain in LC may elucidate additional pathophysiologic mechanisms about the development of NP in LC, and may help guide proper clinical management.

## 2 Methods

### 2.1 Participants

A convenience sample of 153 adults attending a LC clinic at the Icahn School of Medicine at Mount Sinai who reported a history of new-onset pain after acute SARS-CoV-2 infection were included. People attended the clinic if they were: (1) 18 years of age and older; and (2) met criteria for a diagnosis of LC, defined as experiencing new, returning or ongoing health problems 3 months following initial SARS-CoV-2 infection. Participants were excluded from this study if complete data was not available. IRB approval was granted for this study by Mount Sinai STUDY-21-01147.

### 2.2 Measures

#### 2.2.1 Demographics and clinical characteristics

Patients were initially given a clinical intake survey used to characterize their unique LC symptoms that they were currently experiencing or had experienced within the past week. Demographics including sex and age and a detailed symptoms checklist was then extracted from a clinical database.

The Self-Administered Leeds Assessment of Neuropathic Symptoms and Signs (S-LANSS) was used to determine the severity of new-onset pain reported by LC patients and whether it was neuropathic in nature or not. The validated outcomes cut-off value of 12 and above was used (8).

#### 2.2.2 Quality of life measures

The EuroQoL Visual Analog Scale (EQ VAS) and Quality of Life in Neurological Disorders (NeuroQoL) scales were used to assess health-related quality of life (9, 10). The EQ VAS scale is a vertical visual analogue scale that uses values between 100 (best imaginable health) and 0 (worst imaginable health) to provide a broad assessment of health (11). The NeuroQoL is a measurement system that evaluates and monitors the physical, mental, and social effects experienced by adults and children living with neurological conditions (12, 13). The NeuroQoL was used to assess cognitive impairment.

### 2.3 Data analysis

Chi Square and independent *t*-tests were conducted to analyze the relationship between the presence of NP and demographic factors, health-related quality of life measures (results of EQ VAS and NeuroQoL) and other LC symptoms from the symptom checklist. All analyses were performed using SPSS software (version 29.0.2.0).

## 3 Results

### 3.1 Sample characteristics

153 individuals {111 (73%) F, mean (SD) age 46 [(13)] years} with LC who completed the initial survey were included in the analysis. Of these, 31 (20%) were categorized as having NP {26 (84%) F, mean (SD) age 48 [(14)] years}, and 122 (80%) as having non-neuropathic pain (NNP) {85 (70%) F, age 46 [(13)] years} based on their S-LANSS scores. Mean scores of S-LANSS, EQ VAS, and NeuroQoL in each group are reported in Table 1.

**Table 1:** Mean scores of outcome measures in each group.

	Neuropathic pain (*n* = 31)	Non-neuropathic pain (*n* = 122)	Overall	Mean difference	*p*-value
Self-administered leeds assessment of neuropathic symptoms and signs	17.5 (16.1–19.0)	1.8 (1.2–2.4)	5.00 (3.8–6.1)	15.7 (14.4–17.1)	*p* < 0.001
EuroQoL visual analog scale	51.6 (44.1–59.1)	59.8 (56.2–63.4)	58.2 (54.9–61.4)	−8.2 (−16.2 to −0.2)	*p* = .045
Quality of life in neurological disorders	20.8 (18.2–23.5)	25.1 (23.6–26.6)	24.2 (22.9–25.6)	−4.2 (−7.5 to −1.0)	*p* = .011

### 3.2 Most common LC symptoms

The ten most common symptoms in the NNP group were fatigue or tiredness, difficulty with concentration/reading/“brain fog”, dizziness/lightheadedness, memory problems or forgetfulness, headache, difficulty sleeping, shortness of breath, confusion/difficulty thinking, general/muscle weakness and mood swings/irritability/depression. The ten most common symptoms in the NP group were fatigue or tiredness, headache, difficulty with concentration/reading/“brain fog”, memory problems/forgetfulness, difficulty sleeping, confusion/difficulty thinking, general/muscle weakness, shortness of breath, dizziness/lightheadedness, heart palpitations/pulse skips/heart block and muscle pain or cramps. As presented in Table 2, headaches, confusion/difficulty thinking, and generalized muscle weakness were found to be significantly more prevalent in the NP group compared to the NNP group (*p* = 0.01, 0.04, 0.02).

**Table 2:** Frequency and percentage of symptoms experienced by pain group.

Symptom	Neuropathic pain (*n* = 31)	Non-neuropathic pain (*n* = 122)	*p*-value
Fatigue or tiredness	28 (90.3%)	102 (83.6%)	*p* = 0.35
Difficulty with concentration or reading or “brain fog"	26 (83.9%)	87 (71.3%)	*p* = 0.16
Dizziness/Lightheadedness	21 (67.7%)	72 (59.0%)	*p* = 0.37
Memory problems/Forgetfulness	23 (74.2%)	72 (59.0%)	*p* = 0.12
Headache	26 (83.9%)	71 (58.2%)	***p* = 0.01**
Difficulty sleeping	23 (74.2%)	67 (54.9%)	*p* = 0.05
Shortness of breath	21 (67.7%)	66 (54.1%)	*p* = 0.17
Confusion, difficulty thinking	22 (80.0%)	61 (50.0%)	***p* = 0.04**
Generalized muscle weakness	22 (80.0%)	57 (46.7%)	***p* = 0.02**

Significant differences as determined by an independent *t*-test indicated in bold.

### 3.3 Associations between NP, health-related quality of life, and LC symptoms

The NeuroQoL *T*-scores that measure the effects of neurological disorders on quality of life were observed to be significantly lower in the NP group compared to the NNP group (*F* = 0.21, *p* = 0.01). In all participants, NP was associated with the presence of cognitive dysfunction ≥ mild (*χ*^2^ = 5.8, *p* = 0.02). EQ VAS scores were observed to be significantly lower in the NP group compared to the NNP group (*F* = 0.77, *p* = 0.04).

## 4 Discussion

To our knowledge, this is the first study to distinguish between different types of new-onset pain and examine associations between neuropathic pain and cognitive dysfunction in a population with LC. The present study found 31 (20.3%) out of 153 individuals with LC reported NP, which falls within previously reported pooled prevalence range (14).

### 4.1 Presence of symptoms between neuropathic and Non-neuropathic pain groups

**Figure 1:**
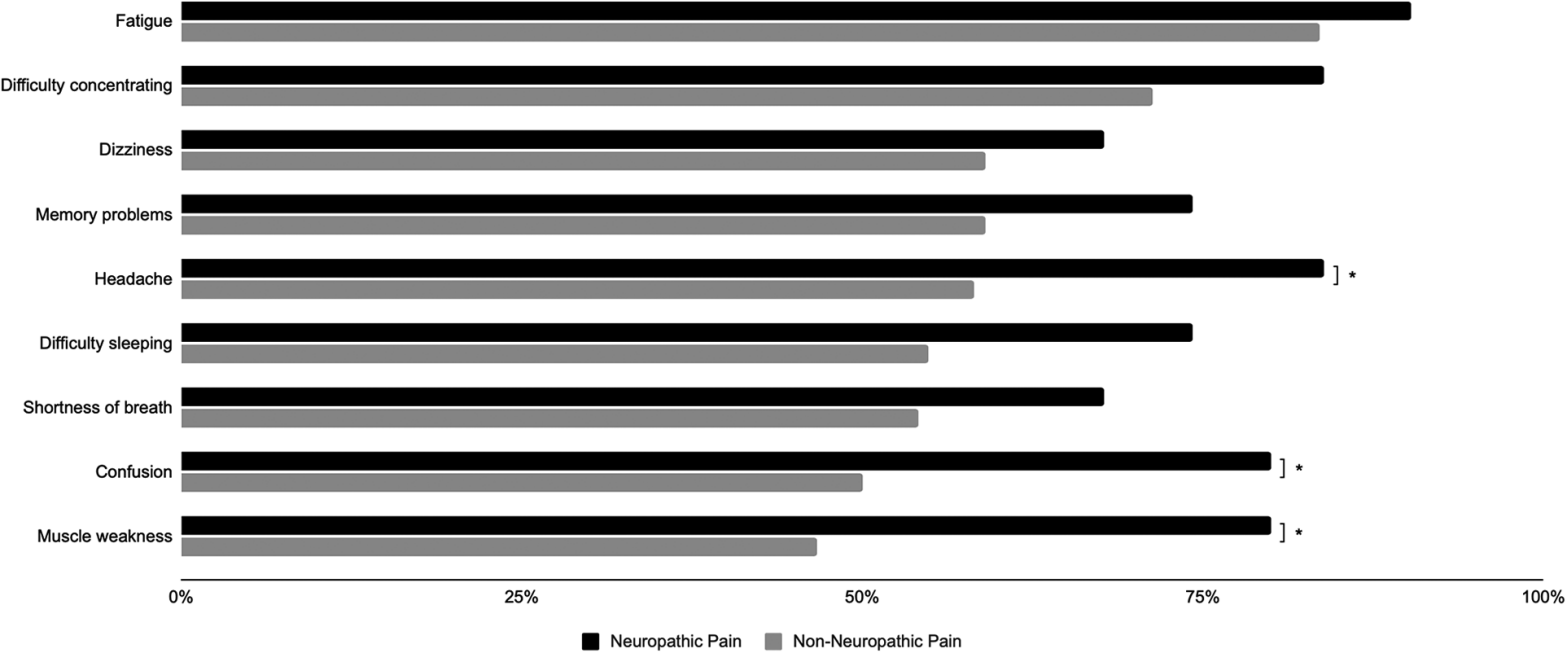
Frequency of symptoms by pain group.

Nine out of the ten most common symptoms were the same among the NNP and NP groups (Figure 1), with only the tenth most common symptom differing between groups. In the NNP group, mood swings/irritability/depression was the tenth most common symptom, whereas heart palpitations/pulse skips/heart block or muscle pains/cramps were more prevalent in the NP group. These overlapping symptoms are consistent with previous literature that has reported “brain fog” and headache as the two most common neurological symptoms of LC, with 85% of participants also experiencing fatigue (15). Headache has also been shown to be a predictor of NP in 90 people with LC, with 4.9 greater odds of having headache after adjusting for age and gender (16). Our present study aligns with this finding that prevalence of headache in LC is higher in those with NP, therefore larger prospective studies should investigate the potential of these symptoms resulting from similar pathophysiology. An understanding of this mechanism may indicate which patients with LC will go on to develop NP.

### 4.2 Pain, cognitive dysfunction, and health-related quality of life

Associations between different types of pain and cognitive dysfunction have been investigated prior to the emergence of LC. It has been established that chronic pain can result in a specific decline in ability to sustain attention (17). The prevalence of cognitive dysfunction has been observed to be substantially higher in patients with NP than those with mixed pain and the general population (18). This work also showed an association between presence of LC-related NP and decreased cognitive function. This aligns with the already established relationship of NP and decline in attention/cognitive function, but adds nuance by specifying that specifically new-onset NP in those individuals with LC is associated with a generalized decrease in cognitive function that may point towards a similar severity in symptoms (19). It is known that cases with higher disease duration or severity of disease are associated with both LC symptoms and specifically NP in LC (19). This study provides further characterization of how debilitating NP can be for those with LC, and illustrates the importance for healthcare providers to continually assess patients pain and cognitive wellbeing, particularly in those who are critically/chronically ill.

This work is amongst the first to show a negative association between quality of life and NP in people with LC. NP has previously been shown to result in quality of life impairment (20). Additionally, LC patients have exhibited diminished cognition-based and fatigue-based quality of life, surpassing demographic expectations (15). Both of these findings align with the findings of this study.

Pain, specifically NP, can be a very distressing sensory experience, resulting in physical, mental and emotional deficits, thereby affecting a patient's ability to carry out activities of daily living. Although it has been proven that pain can cause deficits in attention and memory, it is also possible that pain and cognition have similar neural networks that have yet to be explored.

### 4.3 Mechanisms of pain in long COVID

Past studies have shown depression is a predictor of neuropathic pain in LC, as these symptoms may arise from a common underlying inflammatory process induced by the cytokine storm (19). Additionally, the pain pathway involves the same locations of the brain such as the prefrontal cortex, anterior cingulate and thalamus, all of which play some role in attention and memory (19).

One possible mechanism underlying NP in LC is small fiber polyneuropathy (SFN), which involves preferential damage to thinly myelinated A-delta fibers, un-myelinated C sensory fibers, and autonomic and trophic fibers (21). Symptoms of SFN include chronic pain, sensory impairment, numbness/tingling, and autonomic dysfunction. SFPN has been documented in multiple LC cohorts (21). For example, Oaklander et al. reported SFN in ∼63% of LC participants based on immunohistochemical assessment of lower leg punch biopsies (22).

Research on SFN in patients with diabetes suggests that vasculature and microcirculation issues may contribute to development. Patients with diabetes or pre-diabetes suffer from a high prevalence of SPN, development of which has been linked to impaired vascular endothelial function, impaired microcirculation, platelet hyperactivation, and hypercoagulation (23–27). For example. Pfutzner et al. documented an association between small nerve fiber injury and skin microvascular dysfunction in patients with diabetes mellitus (28). LC is also connected to vascular endothelial dysfunction, platelet hyperactivation, and circulating fibrin/amyloid microclots (29, 30). Thus, the relationship between endothelial and coagulation issues, SFPN, and NP in long COVID requires further investigation. Overall, it is possible that inflammatory and clotting processes in LC blood may limit blood perfusion to the vasa nervorum - the small vessels that deliver blood and oxygen to peripheral nerves, leading to retraction, death, or dysfunctional activity of the energetically demanding peripheral A and C autonomic and pain-transmitting nerve fibers.

SARS-COV-2 has also been shown capable of infecting both the peripheral and central nervous systems, with the long-term persistence of viral RNA or antigen in tissue in nerves reported in some LC cases (31). Thus, SARS-CoV-2 persistence in LC patients with NP pain also requires further investigation, especially since the SARS-CoV-2 spike protein has been shown to seed many clotting and inflammatory processes capable of contributing to neuropathy (32). NP is also common in patients with tickborne/vector borne illness (33). Thus, screening of LC patients for the DNA or RNA of these potential co-infectious organisms such as *Borrelia*, *Bartonella,* or *Babesia* could be warranted.

It is noteworthy that 80% of the present cohort reported new-onset NNP musculoskeletal pain. Although the nature of LC-related musculoskeletal involvement is not well-established, there are many possible factors at play. The main theory describes an elevated inflammatory process caused by SARS-CoV-2 infection on the musculoskeletal and peripheral nerve tissue, where a release of numerous cytokines by the immune system can promote injury and longer-term dysfunction of one or more joints or peripheral nerves. Another theory describes a direct mechanism via SARS-CoV-2 binding to the ACE2 receptor on the skeletal muscle cell surface (34). Persistence of SARS-COV-2 in muscle tissue, ligament or other musculoskeletal tissues could potentially contribute to symptoms in some LC patients. For example, one study found SARS-CoV-2 N protein in LC skeletal muscle tissue, although protein was also found in tissue from controls (33).

### 4.4 Limitations and future implications

It is important to interpret the present findings cautiously due to the constraints of our study design. Most notably, due to the constraints of a limited dataset, generalizing insights is challenging. Nevertheless, the current findings from this study provide novel evidence that the pathophysiology of cognitive dysfunction and NP in LC may be similar. The data also provides a necessary characterization of how detrimental NP due to LC can be on quality of life. Future studies should focus on further establishing associations between NP and demographics and other clinical characteristics including hospital admission and vaccination status, as well as additional instruments to comprehensively evaluate and quantify fatigue, cognitive impairment, and pain. The findings of this research can be used to inform larger studies that appropriately guide treatment and provide insight for management strategies. Most importantly, elucidating the molecular pathophysiology of NP in LC can provide a direct link between NP and other common symptoms in LC.

## Data Availability

The original contributions presented in the study are included in the article/Supplementary Material, further inquiries can be directed to the corresponding author.
